# Silaffins of Diatoms: From Applied Biotechnology to Biomedicine

**DOI:** 10.3390/md11093155

**Published:** 2013-08-26

**Authors:** Igor E. Pamirsky, Kirill S. Golokhvast

**Affiliations:** 1 Analytical Center of Mineralogical and Geochemical Studies, Institute of Geology and Nature Management Far Eastern Branch, Russian Academy of Sciences, 1 Relochny Lane, Blagoveshchensk 675000, Russian Federation; E-Mail: parimski@mail.ru; 2 Laboratory of Nanotoxicology, Engineering School, Far Eastern Federal University, 37 Pushkinskaya street, Vladivostok 690950, Russian Federation

**Keywords:** silaffins, diatoms, silicon, nanomaterials, medicine

## Abstract

Silaffins are involved in the formation of the cell walls of diatoms. It is known that silaffins can precipitate silica *in vitro*, forming nano- and micro-particles in the shape of spheres and plates containing many pores. It is important to note that the deposition of silica and the particle morphology in the presence of silaffins affects chemical and physical agents (e.g., peptides, polyamines, phosphate, nitrogen, and the mechanical changes of the reaction mixture). It is believed that silaffins act as an organic matrix for silica-genesis and that silica pore size should reflect the pattern of a matrix. Here, biotechnology related to silaffins is discussed in the context of “a hypothesis of silaffin matrix” and “the LCPA-phosphate model”. We discuss the most promising area of silaffin biotechnology—the development of production methods for silicon structures with desired shapes and nanostructural properties that can be used to create biocompatible materials.

## 1. Introduction

**Figure 1:**
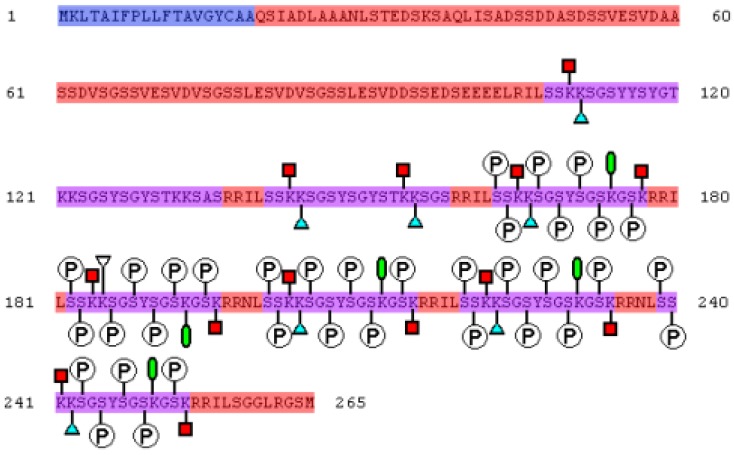
The primary structure of sil1p (a precursor of silaffins) isolated *C. fusiformis*. The amino acid sequence is shown in FASTA format. Silaffins are depicted in violet (natSil-1B 108–136 Am, natSil-1A2 141–158 Am, natSil-1A1 163–177, 182–196, 201–215, 220–234 and 239–253 Am), alarm peptides are shown in blue. The circled letter “P” marks the phosphorylated remains of serine, red squares mark ε-*N*-poly(methylaminopropyl)lysine, blue triangles mark ε-*N*,*N*-dimethyllysine, and green ovals mark ε-*N*,*N*,*N*-trimethyl-5-hydroxylysine.

**Figure 2:**
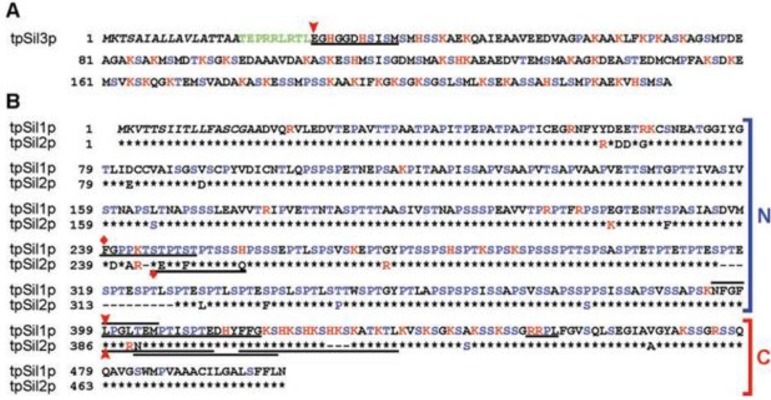
Precursors of silaffins isolated from *T. pseudonana* [10]. Serine, proline and threonine are highlighted in blue, and lysine, arginine and histidine are highlighted in red. The black italics denote alarm peptides. Those discovered by sequencing tryptic *N*-terminal amino acid sequences are emphasized; (**A**) tpSil3p is the precursor of tpSil3. *N*-trailer tpSil3 amino acids are noted by a red arrow. TpSil3 pro-peptide is shown in green; (**B**) for tpSil1p, tpSil1L and tpSil1H are allocated, for tpSil2p, tpSil2L and tpSil2H are allocated. The *N*-and *C*-terminal sites of tpSil1 and tpSil2 are designated by brackets on the right. Identical tpSil1 and tpSil2 amino acids are designated by black asterisks. *N*-trailer sites of tpSil1L and tpSil2L are denoted by a red arrow. The *N*-terminal fragments (35 kDa) of tpSil1/2H are denoted by rhombuses. This research was originally published in The Journal of Biological Chemistry [10]. Copyright © 2004, by the American Society for Biochemistry and Molecular Biology.

Diatoms are the largest group (10,000 types) of unicellular eukaryotic microalgae and are present in virtually all water environments. Diatoms arose 90–280 million years ago. Diatoms are the dominant group of a phytoplankton in the oceans and make up approximately 20%–25% of the general biological primary production on Earth [1,2]. Each type of diatom possesses a silica armor that contains regularly located cracks or pores ranging in size from 10 to 1000 nm [3,4]. Particles of dioxide of silicon (SiO_2_, silicon dioxide), ranging from 10 to 100 nm in diameter, form the basis of a diatom’s cellular wall (epitheca and hypotheca). Orthosilicic acid [Si(OH)_4_] is used to form silicon dioxide and is generally present in the environment at concentrations ranging from tens to hundreds of micromol per liter. Orthosilicic acid arrives into the cell by means of silicon transporter proteins. In the cell, silicic acid, along with special matrix peptides and proteins (e.g., silaffins, silacidins), is turned into amorphous hydrated silicon dioxide. Silaffins, first discovered in the cell walls of diatoms [5], are of special interest for the synthesis of materials with specific properties. Nine varieties of silaffins have been found, and four silaffin genes, isolated from *Cylindrotheca fusiformis* and *Thalassiosira pseudonana* [6,7,8], have been identified. All the silaffins are rich in serine and lysine (Figure 1, Figure 2), and in the course of intracellular maturation are submitted to considerable, and highly similar, post-translational modification [2,9].

Post-translational changes affect most of the amino acid residues, making the chemical structures of mature silaffins extremely complex [6,11,12]. In addition, the *O*-phosphorylation modification stage of serine, threonine, hydroxyproline and hydroxylysine includes monohydroxylation (of lysine and proline), dihydroxylation (proline), glycosylation, sulphation (carbohydrate fragments) and alkylation (ε-amino groups of lysine residues). Lysine-related alkyl groups can be presented by one to three methyl groups or more complex propyleneimin-bearing molecules. The latter modification is closely connected to the formation of long-chain polyamines (LCPA), which consist of linear oligopropyleneimine chains connected with propylenediamine, putrescine or spermidine. Atoms of nitrogen are associated with methylated LCPA in varying degrees, and in certain cases, there are terminal or internal quaternary atoms of nitrogen [2,13].

Research into the mechanism of silaffin action is an important and interesting problem that is of relevance to the fields of biology, materials science, geology and medicine.

## 2. Mechanism of Biomineral Formation

### 2.1. Short Silaffins

**Figure 3:**
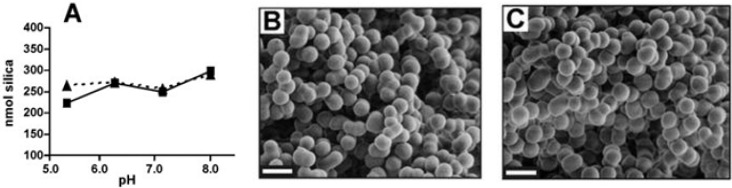
Silicon dioxide precipitated in the presence of silaffins 1A1 and 1A2 [14]; (**A**) Dependence of peptide-induced silicon dioxide genesis on pH. The continuous line (squares) shows the results of the actions of silaffin 1A1, and the dashed line (triangles) indicates the actions of silaffin 1A2. In the photos (scanning electronic microscopy) the silicon dioxide is presented (pH 6.4, 3 mg of peptide on mL) and precipitated by peptides 1A1 (**B**) and 1A2 (**C**). This research was originally published in The Journal of Biological Chemistry [14]. Copyright © 2001, by the American Society for Biochemistry and Molecular Biology.

Silaffins can precipitate silicon dioxide *in vitro* (Figure 3). Silaffins 1A1 and 1A2 precipitate highly similar amounts of silicon dioxide (9.0–11.9 nmol of Si per peptide nmol) at different pH levels [14]. 

A gentle way of extracting native silaffins (using ammonium fluoride at pH 5 rather than hydrogen fluoride, which destroys *O*-glycosides and phosphoesters bonds) was developed, which enabled researchers to establish the important role of residual phosphoric acid in peptide formation [15]. In particular, the numerous groups of phosphates in natSil-1A serve as an internal source of the anions necessary for the formation of silicon dioxide in diatoms. Nuclear magnetic resonance (NMR) research established that the zwitterionic structure of silaffins causes electrostatic interactions between peptides and promotes their self-organization into large units, forming a kind of template for the precipitation of silicon dioxide. Several studies are devoted to researching the ability of R1, R2 and R5 peptides to precipitate silicon dioxide *in vitro* [16,17,18].

**Figure 4:**
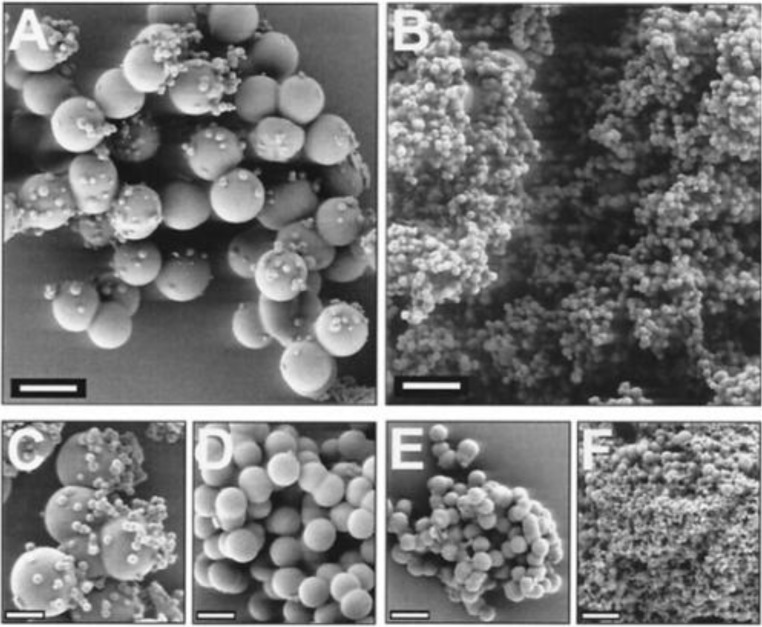
Silicon dioxide precipitated by polyamines of *Nitzschia angularis* [20]. Polyamines with molecular weights ranging from 1000 to 1250 Da (**A**), 600 to 750 Da (**B**), (**C**) and (**F**) Silicon dioxide genesis in the presence of a natural mix of polyamines (molecular weight 600–1250 Da) at different pH levels (**C** pH =5.4, **D** pH =6.3, **E** pH =7.2, **F** pH =8.3). The concentration of polyamines in each solution is 0.85 mg/mL. Bars = 1 µM (**A**, **B**), Bars = 500 nm (**C**–**F**). This figure is reproduced from [20], Copyright © 2000 National Academy of Sciences, USA.

Different factors influence the process of silicon dioxide precipitation in the presence of silaffins, e.g., the presence of gaseous nitrogen, mechanical shifts in the reactionary mix and others [18,19]. Precipitation of silicon dioxide can take place [20] even also on influence of polyamines of modified lysine residues (Figure 4).

**Figure 5:**
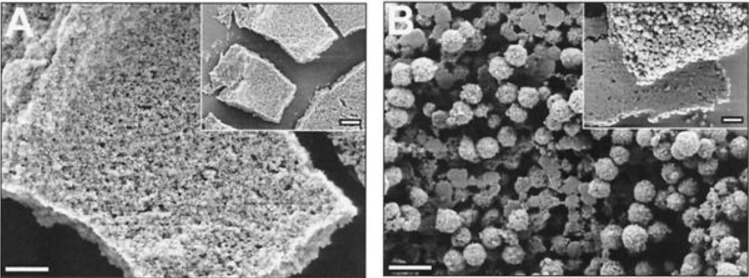
The influence of polyamines isolated from *N. angularis* and silaffins on silicon dioxide morphology [20]; (**A**) Silaffins in concentration of 3 mg/mL; (**B**) Mixed silaffins (3 mL/mL) and polyamines (0.85 mg/mL). Bars = 500 nm (in inserts, bars = 1 µM). This figure is reproduced from [20], Copyright © 2000 National Academy of Sciences, USA.

Polyamines and silaffins together have a synergistic effect (Figure 5) on the morphology of precipitated silicon [20]. Close associations of silaffins and LCPA are capable of physically connecting silicon dioxide molecules by means of electrostatic interactions between silicon and the numerous silaffin amino groups and LCPA or by means of covalent bonds with silicon dioxide [2]. 

**Figure 6:**
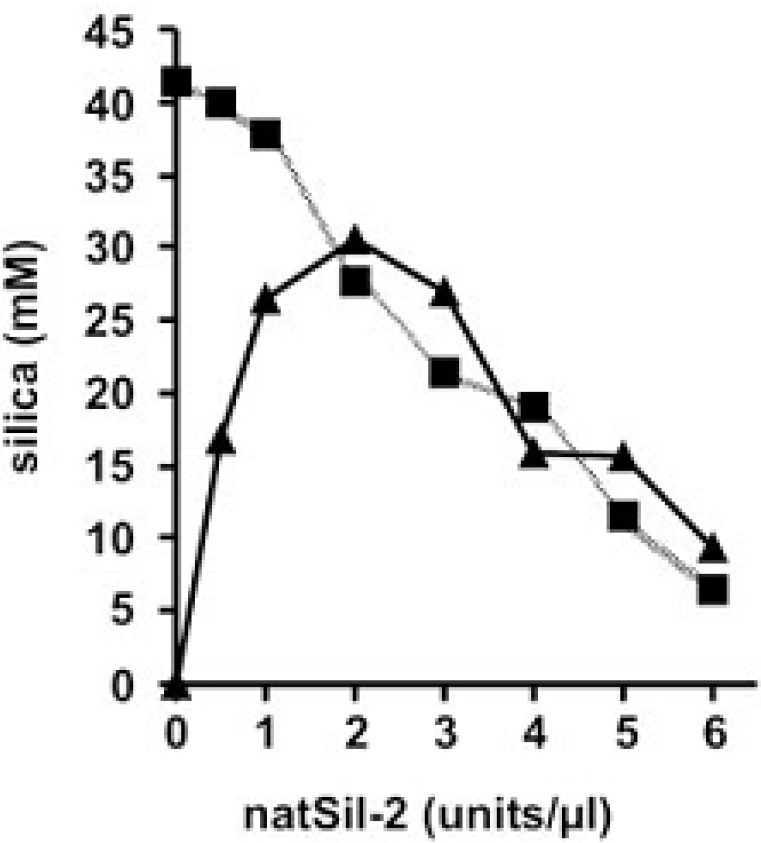
The influence of natSil-2 on silicon dioxide precipitation by a mix of natSil-1A and long-chain polyamines (LCPA) [3]. Precipitation was carried out under normal conditions at constant concentrations of LCPA (0.6 µg/µL; triangles) or natSil-1A (0.3 mm; squares). This figure is reproduced from [3], Copyright © 2003 National Academy of Sciences, USA.

Poulsen *et al.* [3] demonstrated that natSil-2 is incapable of precipitating silicon dioxide from a solution of silicic acid in a test tube, while a mix of natSil-2 and LCPA rapidly formed silicon dioxide precipitates under the same conditions. The authors emphasized that natSil-2 prevented the action of natSil-1A, showing its inhibitory ability on the precipitation of silicon dioxide (Figure 6). 

### 2.2. Long Silaffins

**Figure 7:**
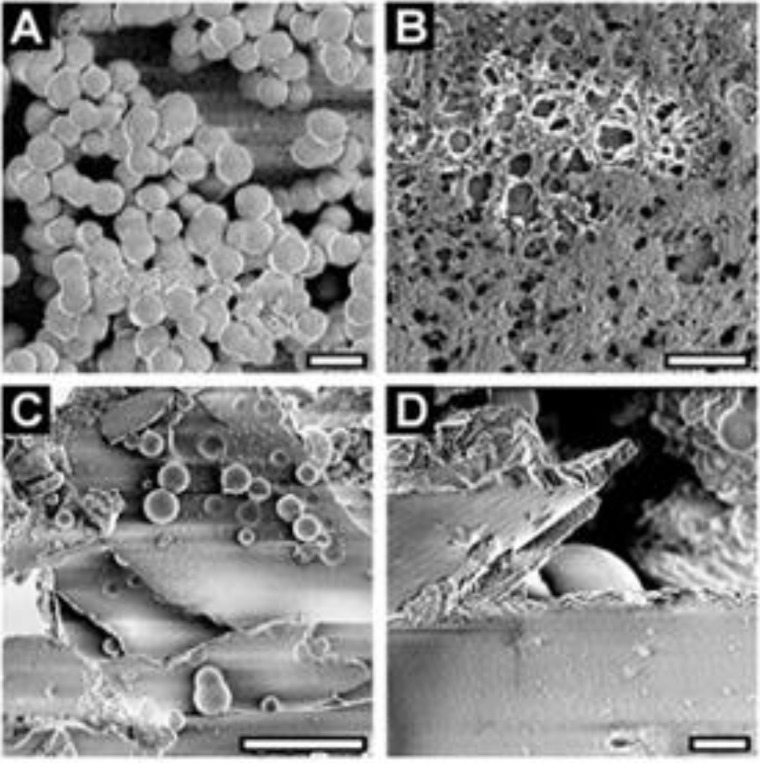
Silicon dioxide morphogenesis in the presence of mixes of silaffins and LCPA [10]. SEM images of (**A**) 20 µM tpSil1/2L (bar = 1 µm); (**B**) 3.5 µM tpSil1/2H (bar = 0.5 µm); (**C**) 20 µm tpSil3 (bar = 10 µm); (**D**) 30 µm tpSil3 (bar = 1 µm). This research was originally published in The Journal of Biological Chemistry [10]. Copyright © 2004, by the American Society for Biochemistry and Molecular Biology.

Post-translational modifications make long-chain silaffins negatively charged. These acidic silaffins can cause *in vitro* formation of silicon dioxide from silicic acid only when combined with LCPA into supramolecular ensembles (Figure 7), which form silicon dioxide even in the absence of phosphate [2,10]. 

Silaffin research has generated “a hypothesis of a silaffin matrix”, which suggests that the interactions of silaffins with LCPA inside of the silica deposition vesicle (SDV) leads to an accumulation of nanostructured organic matrixes with structures supervised by silaffins. Silaffin matrixes act at the same time as an inductor and a template for silicon scurf, preventing silicon dioxide precipitation in silaffin-rich (but LCPA-poor) sites and stimulating silicon dioxide precipitation in LCPA-rich (but silaffin-poor) areas [21]. However, it is unclear how random distributions of LCPA and silaffins are able to stabilize nanostructured silaffin matrixes [2].

Sumper and Bruner [22] presented another model of silicon dioxide morphogenesis based on repeated dispersions of tightly packed LCPA phosphate microdrops, which act as templates for the precipitation of silicon. This “the LCPA phosphate model” is based on a demonstration of the generation of porous structures in a test tube using biomimetic phosphate-polyallylamine systems. Although this model may be important for describing the regularities of the morphogenesis of silicon dioxide with hexagonal pores seen in types of Coscinodiscus, which are not supposed to have silaffins, it does not explain the role of silaffins present in the majority of other diatom types [2]. Another question is raised in this work: if the process of reforming silicon dioxide into nano- and microstructures in the SDV gap depends on a given ratio of silaffin to LCPA, what defines the general form of silicon dioxide structures? In electronic-microscopic research into the formation of silicon dioxide, the connection of SDV with a membrane (silicalemma) is always noted, leading to the assumption that SDV affects the type of silicon formation [23]. The cytoskeleton (microtubules, microfilaments) is closely bound to the cytosolic surface of silicalemma, and impacts to cytoskeleton functions may lead to aberrations in the form of silicon dioxide [23,24,25].

Robinson and Sullivan [26] assumed that the cytoskeleton could also take part in the formation of silicon dioxide nanostructures, thus influencing the formation of germs of silicon dioxide in the SDV gap by transmembrane proteins embedded in the silicalemma. This idea best fits the “a hypothesis of silaffin matrix” model, in which cytoskeleton-connected transmembrane proteins interact with silaffins and stabilize the nanostructured silaffin matrix. Confirmation of this model requires identification of silicalemma proteins that are not obtained due to an absence in the allocation of SDV. A full interpretation of the genome of *T. pseudonana* revealed an alternative way to identify these proteins [2].

## 3. Practical Application of Silaffins

Great attention is paid to silaffins by researchers given the ability of silaffins to initiate and regulate silicon dioxide precipitation at indoor temperature and pressure. Future research into the practical application of similar proteins lies in the development of methods for producing silicon structures with predefined nanostructural forms and properties [27]. Synthesized with the help of silaffins, silicon dioxide is an attractive material for broad application in such areas as macromolecular separation, chemical probing, delivery of drugs, implantology, catalysis, microfluidics, and laboratory chip technologies, *etc.* [27,28,29,30,31,32,33,34,35,36,37,38,39,40,41,42].

Today, chimeric proteins are made using silaffins and other proteins capable of precipitating silicon dioxide in test tubes. For example, from the spidroin 1 domain (MaSp1) of the spider *Nephila clavipes* and the R5 peptide from the silaffin protein of *C. fusiformis* can initiate and regulate precipitation of particles of silicon dioxide 0.5–2 µM in diameter [27]. Additionally, it has been shown that, by regulating the production conditions of polymeric films and fibers, it is possible to supervise the morphology and structure of composites. Another example of such a chimera is that formed from the EAK1 monomer (sequence of *N*-AEAEAKAKAEAEAKAK-*C*), with hydrophobic and polar proteins capable of self-organization, and silaffin (R5 peptide from *C. fusiformis*; sequence of *N*-SSKKSGSYSGSKGSKRRIL-*C*). The chimera of EAK1-R5 is capable of self-assembly in a gel in a test tube in the presence of silicon dioxide [28]. Such chimeric proteins reveal new opportunities of regulating the size of silicon dioxide particles, which provide important advantages during the creation of biomedical materials.

Similar chimeras not only carry out silicon dioxide synthesis but also are used to regulate osteogenesis of human mesenchymal stem cells [29]. Researchers established that the chimera formed from the silk protein from the spider *N. clavipes* and the *C. fusiformis* R5 peptide promoted osteogenic differentiation of mesenchymal stem cells (hMSCs). The presence of silicon from the chimeric protein pellicle (with high adhesion of cells) stimulated the expression of osteogenic genes that were shown to increase the activity of alkaline phosphatase (ALP), as well as bone sialoproteins (BSP) and type 1 collagen (Col 1), in comparison with controls. In two weeks, silicon dioxide calcium scurf (the main component of young apatite) was noted on the pellicle surface.

The creation of biosensors based on chimeras of silaffins appears to be an interesting and promising technology. For example, the hybrid GOX-R5 protein, consisting of R5 and glucose oxidase, was shown to be capable of biosilification in a solution of citric acid (pH 5) and 0.1 M tetramethoxysilane, and can then self-immobilize to form silicon dioxide [30]. Using GOX-R5, the galvanic cell, a biosensor, was developed to control the concentration of glucose in eukaryotic cells. This method of immobilization can be applied to various types of biomolecules.

A simple, universal technique for synthesizing organic and inorganic structures was developed using silaffins and holographic two-photon-induced photopolymerisation (H-TPIP) [31]. Experiments showed that injections of the silaffin-1 peptide (from *C. fusiformis*) together with H-TPIP led to the composition of organic and inorganic structures (polymers of silicon dioxide), which possessed improved optical characteristics (e.g., a fifty times greater diffraction efficiency in lattices containing silicon dioxide) and mechanical properties in comparison to similar polymeric structures without silicon dioxide. Researchers have noted that this method can be applied to the creation of optical devices, for example, antibodies can be included in the hologram for optical identification of specific antigens.

Butyrylcholinesterase and certain other enzymes (data not provided) maintain their activity when immobilized during *in vitro* silicon dioxide bioprecipitation [32]. Approximately 90% (± 7.2) of active butyrylcholinesterase was shown to be immobilized following biosilification in a solution of silicic acid (hydrolyzed tetramethyl orthosilicate) and synthetic R5 peptide (H_2_N-SSKKSGSYSGSKGSKRRIL-COOH, the analogue of the repeating peptide from *C. fusiformis*). The enzymes are immobilized in such a way to be significantly more stable than free enzymes. In subsequent model experiments [33], an easy method of preparing the reactor was formulated based on immobilized enzymes and tested with liquid chromatography. The addition of 6-R5 and R5 peptides (His), butyrylcholinesterase and hydrolyzed tetramethyl orthosilicate (TMOS) to pitch (agarose granules packed into a column and covered with cobalt ions) led to the formation of quartz nanospheres. This matrix had a high affinity for binding enzymes and maintaining their activity for long periods of use. The authors of these works recommend specified methods of immobilization for designing biosensors, which included deactivating systems and reactors. In particular, the use of reactors for screening specific inhibitor enzymes, and for studying their kinetics and potential, is very important developing medicines.

Because of the need to develop more effective methods for the immobilization of active peptides through biosilification, Nam *et al.* [34] synthesized more than 10 chimeric proteins using recombinant DNA technology, which led to successful immobilization in a silicon dioxide matrix rather than adsorption on a silicon dioxide surface. These chimeras, including R5 peptide, the un-modified R1-R7 regions of silaffin from *C. fusiformis* and green fluorescent protein (GFP), showed similar abilities to precipitate silicon dioxide. Similar methods could be applied to immobilize commercially important enzymes for industrial application.

Research into the possible uses of silaffins includes examinations of active enzymes in silicon dioxide from diatoms under natural conditions. For example, the genes encoding hydroxylaminobenzene mutase (HabB from *Pseudomonas pseudoalcaligenes*) were merged with the *C*-terminal end of silaffin tpSil3 [35]. The immobilization of active HabB occurred successfully, with greater than 80% of enzymes maintaining their activity during storage for 30 days at either 48 °C or at freezing. Using scanning electronic microscopy, molecular and genetic manipulations were established to change the silicon dioxide structure of *T. pseudonana*. This method has a number of important advantages, including the following: the proteins do not demand cleaning, the reaction occurs at physiological conditions, and the structures have high mechanical stability, excellent fluidity, and the same ecological safety considerations as silicon dioxide. The authors of this technology see its application as being relative not only to enzymes but also to a wide range of functional proteins (e.g., peptide hormones, growth factors, antibodies, receptors) primarily used as probes or for drug delivery.

Other research into silaffin R5 merged with various functional proteins of *E. coli* (e.g., phosphodiesterase and organophosphorus hydrolase) demonstrated that merged proteins were capable of initiating silicon polycondensation and maintaining enzymatic activity as a part of silicon dioxide [36]. 

Ultrathin pellicles can be created using recombinant silaffin rSilC (17,625 Da) and polycationic matrixes (polyallylamine hydrochloride and polysodium4-styrenesulphonate) [37,38]. The formation of nanoparticles of titan dioxide TiO_2_ can be obtained by adding titanium(IV)-bis(ammonium lactato)-dihydroxide (TiBALDH) to the surface of these pellicle. Other analyses are devoted to the same problem [39]. These technologies will be useful for the creation of hybrid inorganic and organic nanomaterials.

Materials based on silicon dioxide have proved to be catalysts in the degradation of pesticides, electrochemical sensors of NO gas, and optical sensors for combinational dispersion of light (Raman scattering) [2]. The possible use of silaffins as biomarkers of the ecological condition within reservoirs [40] and as gravimetric biosensors [41] is also shown.

It is undoubted that for qualitatively new step in silicon nanobiotechnology it is necessary having studied natural system to create chemical technology similar to it. Except chimeric proteins one of the ways for achievement of this purpose is synthesis of chemical analogs of polyamines [42].

## 4. Conclusions

Any scientific task demands not only serious researches, but also deeply judgment and generalization. And this generalization is useful even at the earliest investigation phases. The biomineralization as process consists of different subprocesses and components which separately as well as whole can be a source of new technologies and materials. 

Silaffins, apparently from the review, already have a wide scope, but bigger part of knowledge about it is still hidden from researchers. For example, the area of medical materials science and bioprosthetics is extremely perspective.

## References

[1] Scala S., Bowler C. (2001). Molecular insights into the novel aspects of diatom biology. Cell. Mol. Life Sci..

[2] Kröger N. (2007). Prescribing diatom morphology: Toward genetic engineering of biological nanomaterials. Curr. Op. Chem. Biol..

[3] Poulsen N., Sumper M., Kröger N. (2003). Biosilica formation in diatoms: Characterization of native silaffin-2 and its role in silica morphogenesis. Proc. Natl. Acad. Sci. USA.

[4] Sumper M. (2002). A phase separation model for the nanopatterning of diatom biosilica. Science.

[5] Kröger N., Deutzmann R., Sumper M. (1999). Polycationic peptides from diatom biosilica that direct silica nanosphere formation. Science.

[6] Kröger N., Poulsen N. (2008). Diatoms—From cell wall biogenesis to nanotechnology. Annu. Rev. Genet..

[7] Pamirsky I.E., Golokhvast K.S. (2012). Search for homologues of proteins of primitive organisms biomineralization. Achiev. Life Sci. Russ..

[8] Golokhvast K.S. (2010). Interaction of Organisms with Minerals.

[9] Sumper M., Kröger N. (2004). Silica formation in diatoms: The function of long-chain polyamines and silaffins. J. Mater. Chem..

[10] Poulsen N., Kröger N. (2004). Silica morphogenesis by alternative processing of silaffins in the diatom *Thalassiosira pseudonana*. J. Biol. Chem..

[11] Sumper M., Hett R., Lehmann G., Wenzl S. (2007). A code for lysine modifications of a silica biomineralizing silaffin protein. Angew. Chem. Int. Ed..

[12] Wieneke R., Bernecker A., Riedel R., Sumper M., Steinem C., Geyer A. (2011). Silica precipitation with synthetic silaffin peptides. Org. Biomol. Chem..

[13] Gröger C., Lutz K., Brunner E. (2008). Biomolecular self-assembly and its relevance in silica biomineralization. Cell. Biochem. Biophys..

[14] Kröger N., Deutzmann R., Sumper M. (2001). Silica-Precipitating peptides from diatoms. The chemical structure of silaffin-1a from *Cylindrotheca fusiformis*. J. Biol. Chem..

[15] Kröger N., Lorenz S., Brunner E., Sumper M. (2002). Self-Assembly of highly phosphorylated silaffins and their function in biosilica morphogenesis. Science.

[16] Patwardhan S.V., Shiba K., Schroder H.C., Muller W.E.G., Clarson S.J., Perry C.C. (2007). The interaction of silicon with proteins: Part 2. The role of bioinspired peptide and recombinant proteins in silica polymerization. Sci. Technol. Silicones Silicone-Modif. Mat..

[17] Whitlock P.W., Patwardhan S.V., Stone M.O., Clarson S.J., Cheng H.N., Gross R.A. (2008). Polymer Biocatalysis and Biomaterials II.

[18] Wong Po Foo C., Huang J., Kaplan D.L. (2004). Lessons from Seashells: Silica mineralization via protein templating. Trends Biotechnol..

[19] Patwardhan S.V., Clarson S.J. (2002). Silicification and biosilicification. Part 4. Effect of template size on the formation of silica. J. Inorg. Organomet. Polym..

[20] Kröger N., Deutzmann R., Bergsdorf C., Sumper M. (2000). Species-Specific polyamines from diatoms control silica morphology. Proc. Natl. Acad. Sci. USA.

[21] Kröger N., Poulsen N., Bäuerlein E. (2007). Handbook of Biomineralization.

[22] Sumper M., Brunner E. (2006). Learning from diatoms: Nature’s tools for the production of nanostructured silica. Adv. Funct. Mater..

[23] Pickett-Heaps J., Schmid A.M.M., Edgar L.A., Round F.E., Chapman D.J. (1990). Progress in Phycological Research.

[24] Van De Meene A.M.L., Pickett-Heaps J.D. (2002). Valve morphogenesis in the centric diatom *Proboscia alata* Sundstrom. J. Phycol..

[25] Tesson B., Hildebrand M. (2010). Dynamics of silica cell wall morphogenesis in the diatom *Cyclotella cryptica*: Substructure formation and the role of microfilaments. J. Struct. Biol..

[26] Robinson D.H., Sullivan C.W. (1987). How do diatoms make silicon biominerals?. Trends Biochem. Sci..

[27] Wong Po Foo C., Patwardhan S.V., Belton D.J., Kitchel B., Anastasiades D., Huang J., Naik R.R., Perry C.C., Kaplan D.L. (2006). Novel nanocomposites from spider silk-silica fusion (chimeric) proteins. Proc. Natl. Acad. Sci. USA.

[28] Marner W.D., Shaikh A.S., Muller S.J., Keasling J.D. (2008). Morphology of artificial silica matrices formed via autosilification of a silaffin/protein polymer chimera. Biomacromolecules.

[29] Mieszawska A.J., Nadkarni L.D., Perry C.C., Kaplan D.L. (2010). Nanoscale control of silica particle formation via silk-silica fusion proteins for bone regeneration. Chem. Mater..

[30] Okkyoung C., Byung-Chun K., Ji-Hye A., Kyoungseon M., Yong H.K., Youngsoon U., Min-Kyu O., Byoung-In S. (2011). A biosensor based on the self-entrapment of glucose oxidase within biomimetic silica nanoparticles induced by a fusion enzyme. Enzym. Microb. Technol..

[31] Brott L.L., Naik R.R., Pikas D.J., Kirkpatrick S.M., Tomlin D.W., Whitlock P.W., Clarson S.J., Stone M.O. (2001). Ultrafast holographic nanopatterning of biocatalytically formed silica. Nature.

[32] Luckarift H.R., Spain J.C., Naik R.R., Stone M.O. (2004). Enzyme immobilization in a biomimetic silica support. Nat. Biotechnol..

[33] Luckarift H.R., Johnson G.R., Spain J.C. (2006). Silica-Immobilized enzyme reactors; Application to cholinesterase-inhibition studies. J. Chromatogr..

[34] Nam D.H., Won K., Kim Y.H., Sang B.I. (2009). A novel route for immobilization of proteins to silica particles incorporating silaffin domains. Biotechnol. Prog..

[35] Poulsen N., Berne C., Spain J., Kroger N. (2007). Silica immobilization of an enzyme through genetic engineering of the diatom *Thalassiosira pseudonana*. Angew. Chem. Int. Ed..

[36] Marner W.D., Shaikh A.S., Muller S.J., Keasling J.D. (2009). Enzyme immobilization via silaffin-mediated autoencapsulation in a biosilica support. Biotechnol. Prog..

[37] Kharlampieva E., Jung C.M., Kozlovskaya V., Tsukruk V.V. (2010). Secondary structure of silaffin at interfaces and titania formation. J. Mat. Chem..

[38] Kharlampieva E., Slocik J.M., Singamaneni S., Poulsen N., Kroger N., Naik R.R., Tsukruk V.V. (2009). Protein-Enabled synthesis of monodisperse titania nanoparticles on and within polyelectrolyte matrices. Adv. Funct. Mat..

[39] Sewell S.L., Wright D.W. (2006). Biomimetic synthesis of titanium dioxide utilizing the R5 peptide derived from *Cylindrotheca fusiformis*. Chem. Mat..

[40] Carvalho R.N., Burchardt A.D., Sena F., Mariani G., Mueller A., Bopp S.K., Umlauf G., Lettieri T. (2011). Gene biomarkers in diatom *Thalassiosira pseudonana* exposed to polycyclic aromatic hydrocarbons from contaminated marine surface sediments. Aquat. Toxicol..

[41] Nam D.H., Lee J.-O., Sang B.-I., Won K., Kim Y.H. (2013). Silaffin peptides as a novel signal enhancer for gravimetric biosensors. Appl. Biochem. Biotechnol..

[42] Annenkov V.V., Patwardhan S.V., Belton D., Danilovtseva E.N., Perry C.C. (2006). A new stepwise synthesis of a family of propylamines derived from diatom silaffins and their activity in silicification. Chem. Commun..

